# Emergency Services During the SARS-CoV-2 Pandemic: A Gender Comparison of Burnout Risk and Personality Traits in the Kharkiv City Sample

**DOI:** 10.3390/healthcare12232356

**Published:** 2024-11-25

**Authors:** Igor Zavgorodnii, Beatrice Thielmann, Olena Litovchenko, Victor Zabashta, Valerij Kapustnyk, Robin Schwarze, Irina Böckelmann

**Affiliations:** 1 Department of Hygiene and Ecology No 2, Kharkiv National Medical University, 61022 Kharkiv, Ukraine; zavnikua@gmail.com (I.Z.); latyshkaelena@gmail.com (O.L.); 2 Institute of Occupational Medicine, Faculty of Medicine, Otto von Guericke University, 39120 Magdeburg, Germany; robin.schwarze@mail.de (R.S.); irina.boeckelmann@med.ovgu.de (I.B.); 3 Communal Non-Commercial Enterprise of the Kharkiv Regional Council “Center for Emergency Medical Care and Disaster Medicine in the Kharkiv Region”, 61058 Kharkiv, Ukraine; ekstrena.dopomoga@ukr.net; 4 Department of Internal and Occupational Diseases, Kharkiv National Medical University, 61166 Kharkiv, Ukraine; kapustnik.valerij@ukr.net

**Keywords:** emergency services, COVID-19, burnout, personality traits, emotional exhaustion, cynicism

## Abstract

**Introduction**: The SARS-CoV-2 pandemic presented unique challenges to the health-care system and prehospital emergency medical services. An increasing prevalence of burnout has been described, which in turn is associated with mental illness. The aim of this paper was to evaluate burnout through a sex comparison and to analyze associations of burnout with personality traits during the SARS-CoV-2 pandemic. **Methods**: Eighty-eight emergency physicians and field shearers of Kharkiv City (Ukraine) emergency medical services (52% women) participated in the quantitative cross-sectional study. In addition to sociodemographic and occupational data, the Maslach Burnout Inventory (MBI) and the Freiburg Personality Inventory (FPI) were applied and analyzed in the sex comparison. Correlation analyses were performed to describe the relationships between the MBI dimensions and FPI traits. **Results**: The average age of the respondents was 35.1 ± 13.5 years. The prevalence of burnout during the pandemic was 6.5% in women and 2.4% in men. Only the scores on the MBI dimension cynicism were significantly (*p* = 0.027) higher in women than in men. Two personality traits differed between sexes: inhibition and male/female self-reports. Predominantly moderate correlations were found between the FPI traits and the MBI dimensions. **Conclusions**: Although the prevalence of burnout in this occupational group during the pandemic was similar to prepandemic figures reported in the literature, more than half of the male and female paramedics showed average-to-high scores on the three MBI dimensions. Because burnout is associated with other mental illnesses and prolonged incapacity, workplace-based interventions should be implemented.

## 1. Introduction

After the new SARS-CoV-2 variant emerged for the first time in Wuhan (China) in autumn 2019 and the consequent worldwide outbreak of COVID-19, the WHO declared the international health emergency to be a pandemic in March 2020. On 5 May 2023, the WHO lifted the global coronavirus health emergency after a total of 3 years. Especially in the first two years of the pandemic, health-care workers experienced an exceptionally high level of stress, which was characterized by worry and an increase in physical and psychological strain [1]. Many studies from the prepandemic period describe burnout syndrome as a possible long-term consequence of coping with the demands of work and the persistence of short-term effects caused by psychological stress [2,3,4,5].

Burnout syndrome is more likely to be suspected in individuals who have high emotional exhaustion and cynicism/depersonalization and low performance (professional efficiency) [6,7,8]. Research shows that occupational burnout can have serious personal and professional consequences for individuals and is an important health economic factor for society [9,10,11]. There are a variety of risk factors for burnout, such as structural/organizational, psychological/emotional, environmental, or sociodemographic factors [12]. Identified risk factors that promote burnout syndrome include increased workload, work compression, a lack of self-determination, self-control, and low professional experience, as well as rewards, a breakdown of the sense of community, a lack of fairness, value conflicts, and poor work–life balance [13,14,15]. Employee satisfaction increases with sufficient opportunities to influence the organizational level, access to resources and rewards, and a supportive and equitable social environment. Deficits in these areas promote the development of burnout symptoms. Additional factors and personality traits that play a role in ensuring that not every person who is exposed to high levels of job stress develops burnout have not yet been conclusively determined. Individuals with personality traits such as inhibition; emotionality, in the sense of mood lability; and irritability have fewer resources to cope with stress and therefore have an increased risk of burnout [15]. A complex interaction between having a dysfunctional, closed-off personality and burnout has been described [16].

The changed stress situation as a result of the pandemic and the resulting stresses have been described in international emergency services [17,18,19,20,21,22]. Little research data on the prevalence of burnout risk among emergency medical personnel in Ukraine are available. In this study, in the prepandemic period (2018), it was shown that male subjects in the ambulance service of the Ukrainian city of Kharkiv had a significantly higher risk of burnout than female subjects (7.1% vs. 1.8%) [23]. No sex-related differences in the degree of emotional exhaustion or performance were found. However, of the male paramedics, 60.7% had significantly higher scores on the cynicism/depersonalization dimension compared to the female paramedics. First studies show that the pandemic increases the prevalence of burnout risk [18,24,25,26]. Whether there are indeed gender differences in the expression of burnout symptoms among emergency service workers in Ukraine remains to be investigated.

The psychological stress situation of the emergency medical services is well known [27].

The study was conducted to assess whether pandemic-induced stress and changes in working conditions affected burnout risk among emergency medical service (EMS) personnel in Kharkiv, Ukraine, and to explore the influence of personality traits on burnout dimensions. There is still a considerable need for research in relation to employees in the EMSs. Based on the working hypotheses, the study aimed to determine whether gender differences exist in burnout risk and personality traits among EMS personnel and to analyze the correlation between these factors during the SARS-CoV-2 pandemic.

## 2. Materials and Methods

This work was based on a quantitative paper–pencil cross-sectional study in which data collection took place by means of standardized questionnaires. The questions were answered independently and anonymously, and the questionnaire was handed back in closed form. The Ukrainian cooperation partners were responsible for sample recruitment and the distribution of paper questionnaires. Study participation was voluntary. Ambulance staff were informed by the respective heads of the stations. If consent was given, the study-group staff went directly to each ambulance station and conducted a group briefing. At the same time, medical staff were made aware of the aims and objectives/questions of the project, as well as the rules and deadlines for completing the questionnaires. Ambulance staff of the Charkow region completed surveys from 28 May to 7 June 2021. In March 2021, the third wave of the coronavirus pandemic began in Ukraine, peaking in October 2021. Thus, this survey took place at the beginning of the third wave. The inclusion criterion was a full-time job for at least one year in the emergency medical service. Exclusion criteria were therefore a part-time job or less than one year of work experience.

### 2.1. Participants

The Ukrainian emergency medical services are described in more detail below, as there are no standardized regulations, and emergency medical services vary greatly from country to country. In Ukraine, a distinction is made between an emergency medical team and a paramedic team. An emergency medical team consists of an emergency physician, a field shearer, a nurse, and a driver. A paramedic team consists of a field shearer, nurse, and driver. The drivers are not involved in medical treatment [28]. In Ukraine, the specialist designation of an emergency physician is obtained after the successful completion of six years of study in human medicine at a medical school institution, followed by 1.5 years of an internship in an accredited emergency medical service institution or in the regional center for emergency medical care and disaster medicine. Emergency physicians are used only in specific, severe cases, as is also standard in many other industrialized countries [28]. A field shearer is the nonphysician leader of a paramedic team who is also personally responsible for the team’s work. Since 2018, it has been possible to train academically as a paramedic in Ukraine over three years (completion of at least a bachelor’s degree). The qualification requirements for a paramedic are higher than those for a field shearer because the training program for a paramedic includes emergency care to a greater extent [28]. The Ukrainian paramedic service is organized, coordinated, and controlled by the Cabinet of Ministers of Ukraine and is part of the emergency care system. This includes emergency care and disaster medical centers, emergency stations, and an emergency medical assistance department. Emergency medical stations are subdivided into dispatch services, mobile teams, and administration. Mobile teams provide care and treatment to the local population and transport patients to medical facilities. The major city of Kharkiv has nine state-funded ambulance stations, each of which has up to 10 mobile teams. Even during the pandemic period, there was no shortage of skilled workers, as these professionals are well paid compared to the general population and enjoy a very high reputation among the population [28]. The type of team was not considered in the survey. All professional groups in the medical emergency services were surveyed.

### 2.2. Methods

The sociodemographic questionnaire asked the usual questions about individuals, such as age, sex, marital status, and the number of children living in the household or family members in need of care. In addition, data on occupation-related variables were recorded, such as profession (emergency physicians and field shearers), work experience, managerial or nonmanagerial position, working hours (as a grouped variable), and weekend work.

The *Maslach Burnout Inventory—General Survey (MBI-GS)* determines burnout severity using 16 items covering the hypothesized aspects of burnout syndrome that are grouped into the three dimensions of “emotional exhaustion”, “cynicism”, and “performance” (professional efficiency) [29,30]. The possible answers vary and are scored on a seven-point Likert scale from “never” (0) to “daily” (6). By calculating the mean values of the respective dimensions, the expression of the three burnout dimensions can be assessed. The “efficiency” dimension addresses the respondent’s assessment of their own professional ability or their perception of professional self-efficacy. This dimension maps professional efficiency. If the scores on the “emotional exhaustion” and “cynicism” dimensions are high and the score on the “performance” dimension is low, an increased risk for burnout syndrome can be concluded.

To better classify the mean scores on each MBI dimension, Maslach et al. [29] used the values from a North American sample (*n* = 3727) to form three degrees of burnout: low, average, and high.

To be able to make a precise statement regarding the individual risk of burnout, the classification of burnout risk according to Kalimo et al. [31] was applied.

The *Freiburg Personality Inventory (FPI)* was designed to assess personality traits that are fundamental to social adjustment and behavioral control. The inventory consists of 114 items and captures personality with 12 factors [32]. These 12 factors are nervousness, spontaneous aggressiveness, depressiveness, excitability, sociability, composure, reactive aggressiveness, inhibition, openness, extraversion, emotional lability, and masculinity. On a two-point response scale, respondents agree (“agree” (1)) or disagree (“disagree” (2)) with the statements. In a second step, these primary assessments are converted into standard assessments scored on a nine-point scale using a special table. Low scores range from 1 to 3 points, medium scores range from 4 to 6 points, and high scores range from 7 to 9 points.

### 2.3. Statistical Analysis

The sample size was determined according to Cohen 1988 [30]: Cohen’s d of 0.8, alpha level of 0.05, power of 0.8, and two-sided testing. With an effect size of d = 0.8 and a power of 0.8, 26 subjects would be needed per group (52 in total).

The data were analyzed by applying the Statistical Package of Social Science (SPSS version 28.0, New York, NY, USA) statistical program. First, the data were subjected to descriptive analysis to determine the frequency, mean (MW), standard deviation (SD), median, range (minimum (Min)–maximum (Max)), and 95% confidence interval (95% CI). Since the data from the Kharkiv sample were not normally distributed, non-parametric tests were used. For the mean comparisons of both sex groups, the nonparametric Mann–Whitney U test for independent samples was applied. The chi-square test and Fisher’s exact test were used to calculate the significance of the frequency differences. A significance level of *p* < 0.05 was set for all calculations. To determine correlations between the personality traits and burnout dimensions, an additional Spearman correlation analysis was performed. Spearman’s rho (ρ) effects were assessed according to Cohen (1988) as follows: ρ < 0.1 indicated no effect, ρ = 0.1–0.29 indicated a weak effect, ρ = 0.300 to 0.499 indicated a medium effect, and ρ ≥ 0.500 indicated a strong effect [33].

## 3. Results

### 3.1. Sociodemographic and Occupation-Related Data

The sample consisted of a total of 88 employees (39 emergency physicians and 49 field shearers) of Kharkiv emergency medical services (EMSs) (46 women, 42 men). The mean age of the respondents was 35.1 ± 13.5 years (median 30 years; minimum 19 years; maximum 67 years).

The sociodemographic and occupational data are shown in Table 1, Table 2 and Table 3.

The sociodemographic data of both sexes were statistically comparable (Table 3).

### 3.2. Results of MBI

The results of the MBI-GS for the total sample of rescue personnel are shown in Table 4, taking sex into account. The average emotional exhaustion score of the total sample was 2.61 ± 1.612 points, and emotional exhaustion was thus pronounced (average degree of emotional exhaustion ranged from 2.01 to 3.19). The two sex groups did not differ significantly from each other (*p* = 0.572).

On average, the cynicism/depersonalization dimension was also pronounced. With a value of 1.76 points, the value for the total sample was within the range of the average score (between 1.01 and 2.19 points). The degree of cynicism was significantly (*p* = 0.042) higher among the men surveyed (2.01 ± 1.25 points) than among the women surveyed (1.54 ± 1.46 points).

There was no significant difference in the performance dimension between the two sex groups (*p* = 0.217). The mean score of the total sample was 5.07 ± 1.208 points and could thus be classified as high.

The MBI total scores for the male (1.89 ± 0.94) and female (1.82 ± 1.014) respondents were statistically comparable (*p* = 0.764) and, according to the classification of Kalimo et al. [31], in the range of “some burnout symptoms”.

The distribution of subjects with different scores on the MBI dimensions (“low”, “average”, and “high”) in the total sample and in the sex groups is shown in Table 5. Every other man showed a high (≥2.20) score on the MBI “cynicism” dimension, whereas in the group of women, only 28.3% had a high “cynicism” score. These differences were significant (*p* = 0.027). At the same time, 47.8% of the female respondents had lower scores on the MBI “cynicism” dimension (≤1.00). In the male-sex group, only 21.4% had lower scores in this dimension.

The risk of burnout was assessed on the basis of the MBI total score. The distribution of the subjects with different MBI total scores according to Kalimo et al. [25] (“no burnout”, “some burnout symptoms”, and “burnout risk”) in the total sample and in the sex groups is shown in Table 5. There were no significant differences between the sex groups (*p* = 0.523).

The prevalence of burnout risk was found to be 4.5% among all employees. For women, the prevalence was slightly higher, but this was not statistically significant.

### 3.3. Results of FPI

The evaluation of various categories of personal qualities by sex showed (Table 6) that most indicators were within the normal range. At the same time, “inhibition” was statistically significantly (*p* = 0.025) lower among the surveyed men (5.60 ± 1.754 points) than among the surveyed women (6.54 ± 1.486 points).

### 3.4. Relationship Between Personality Traits and Burnout

In the study sample, significant correlations were found between personality traits and emotional exhaustion, as well as cynicism (Table 7). Strong correlations were found for nervousness and depressiveness, as well as for the MBI total score (ρ = 0.521 and *p* = 0.511, respectively).

It can be seen in Table 7 that the burnout dimension performance did not correlate with any personality trait. Additionally, some personality traits (sociability, extraversion, and masculine/feminine depiction) did not show any correlation with the individual burnout dimensions.

## 4. Discussion

This study examined the relationship between the burnout risk and personality traits of emergency physicians and field nurses of the State Emergency Medical Service in Kharkiv, the second largest city in Ukraine, during the third wave of the SARS-CoV-2 pandemic. The focus was on a sex-specific approach. The sample was homogeneous in terms of sociodemographic and occupational information. Scores on the MBI dimensions were average in severity and differed only with respect to cynicism. Here, men were significantly more cynical about their work than women. The prevalence of burnout was 4.5% for the total sample in this study. The results of the survey are similar to those of a comparable prepandemic sample [23]. Interestingly, the prevalence changed within the sex groups. Prior to the pandemic, 7.1% of male and 1.8% of female EMS workers showed symptoms of burnout [23]. The survey during the third pandemic wave in Ukraine showed that 6.5% of females and 2.4% of males showed burnout symptoms; however, due to the smaller sample, this statement cannot be generalized.

Regarding FPI personality traits, significant differences were found only for the traits of “inhibition” (women > men) and “male/female self-description” (women < men). High test scores for “inhibition” indicated shyness, inhibition in dealing with others, the inability to make contact, stage fright and physical discomfort before certain occasions, low drive, and a low willingness to assert oneself. High test scores for “male/female self-description” represented, for example, active assertiveness, self-confidence, a balanced mood, and little physical discomfort [34]. These two personality traits may serve as a basis for reversing burnout risk among Ukrainian male and female paramedics. Other reasons for the altered scores on the MBI dimensions may lie in the increased family burden on women. In Ukraine, kindergartens and schools were also closed during the pandemic period. As a result, online teaching increased, and food was provided at home (no school lunch). From the literature, we can see that sex differences in the stress response increase [35] susceptibility to stress-related mental disorders [36,37]. In addition, differences in the symptomatic expression of the same mental health problem have been described [38]. A meta-analysis showed that female sex was a risk factor not only for burnout but also for posttraumatic stress disorder during the pandemic [39].

The correlation analysis of our data showed predominantly moderate correlations between the FPI personality traits and the MBI dimensions of emotional exhaustion and cynicism, as well as the MBI total score. Here, increases in the scores for the personality traits “nervousness”, “depressiveness”, and “emotional lability” were associated with increases in the scores on the MBI dimensions of emotional exhaustion and cynicism. Similar associations were shown in the study by Bergmueller et al. [40]. These personality traits also correlated more strongly with the MBI dimensions than the other FPI personality traits during the pandemic. In a study with Ukrainian bank employees, there were medium positive correlations between emotional exhaustion in the MBI and nervousness, depressiveness, and emotional instability in the FPI. Medium correlations were also found between cynicism/depersonalization in the MBI and aggressiveness, emotional instability, and depressiveness [41].

Overall, the prevalence of burnout among Ukrainian paramedics during the pandemic appears to be lower compared to that in individuals in other professions and countries. Among Spanish radiologists, the prevalence of burnout was 49.3%, but the time of the survey differed (04/20–08/20), and the MBI-HSS, a variant of the MBI, was applied without using Kalimo’s classification. A survey from March to August 2020 showed a burnout prevalence of 24% among Singaporean health workers [42]. The extent to which survey timing plays a role in burnout prevalence remains debatable. High prevalences of burnout were seen primarily in the early phases of the pandemic. During this time, there was little knowledge about the coronavirus and its effects, personal protective equipment was inadequate, and vaccinations against the virus were not yet available. Similarly, among the respondents, SARS-CoV-2 infection status was mostly unknown. It has been described in the literature that SARS-CoV-2 infection can be associated with mental disorders [43].

Looking at the values of the individual MBI dimensions during the pandemic in comparison to the prepandemic period, it can be seen that the mean values of the two dimensions of emotional exhaustion and cynicism decreased for men and increased for women. For performance, the reverse was observed [23].

Emergency-department personnel were in direct contact with known or even unknown SARS-CoV-2 patients during emergency operations. A study from Italy showed that hospital staff in COVID-19 units, where staff had direct contact with SARS-CoV-2 patients, exhibited more emotional exhaustion and cynicism than staff in non-COVID-19 units [44]. It should be noted, however, that for ambulance personnel, patient contact does not always last for the entire duration of the work shift, as is the case for hospital personnel. Nevertheless, there may be a fear of possible infection due to contact with patients with an unknown infection status. The results of a study of physicians from the United States showed that physicians experienced more stress and burnout when they had to work with unvaccinated patients or colleagues [45]. A review also documented that health-care workers exposed to COVID-19 had a significantly higher prevalence of mental health problems [46].

A study from Spain also showed an increase in emotional exhaustion and burnout among physicians and nurses in public emergency departments during the pandemic [47]. Similar trajectories were seen among dental health-care personnel [48] and Turkish nursing staff in “pediatric emergency departments” [49]. A meta-analysis including 16 studies reported a prevalence of 34.1% for emotional exhaustion, 12.6% for cynicism/depersonalization, and 15.2% for impaired performance among nurses. However, the meta-analysis did not allow conclusions to be drawn regarding the survey instrument used to assess burnout risk in each study [50]. Our data showed partially higher values for the scores of individual dimensions. A total of 37.5% of the total sample indicated emotional exhaustion (women 41.3%; men 33.3%), and 38.6% indicated cynicism (women 28.3%; men 50%). Studies show that these burnout dimensions are associated with other mental illnesses during the pandemic, such as depression [51] or anxiety [52]. Various reviews have shown that the abovementioned mental illnesses were more prevalent among frontline health-care workers than among other health-care workers [39,53]. This included emergency medical services personnel.

The US study showed that the security measures implemented during the COVID-19 pandemic had a significant impact on the working environment of paramedics, with 51% reporting increased conflicts between colleagues [54]. Higher levels of stress were observed among those who reported conflict, and paramedics were more likely to experience these tensions than emergency medical technicians. Mandatory overtime policies were strongly associated with increased workplace conflict, highlighting the need to address such stressors in future interventions [54].

Therefore, it is relevant to identify factors of burnout from an occupational health perspective and to counteract it [22]. From the perspective of personnel development and safety needs, it is relevant for managers to perceive and address symptoms of emotional exhaustion and, above all, take symptoms expressed by employees seriously and counteract them [55]. This is particularly important for occupational groups that are exposed to a VUCAH environment [56]. This includes emergency services, as they are characterized by high volatility, complexity, uncertainty, ambiguity, and interconnectedness [56]. Incapacity days in all occupational sectors increased by more than 50% within one decade (2012–2021) [57]. Data on incapacity to work due to mental illness from Ukraine are not available.

In terms of occupational medicine, a preventive approach, i.e., the implementation of preventive measures in a phase that is perceived as healthy with risks for burnout, is relevant here [58,59]. This is even more important given that, in addition to burnout, high prevalences of post-traumatic stress disorder, depression, anxiety, and high-risk alcohol use have been reported in EMS personnel [60]. In this context, it is important to perceive and use signals from the body and the soul to bring about change. Symptoms such as shortness of breath, dizziness, digestive problems, difficulty sleeping, irritability, or difficulty concentrating can indicate the presence of burnout [61]. Framework conditions should be adjusted, and priorities should be established. The development of working relationships in a trusting manner ensures the psychological stability of employees. Useful questions here can include the following: What am I missing? Which work tasks are currently (still) unfulfillable? Where do I need support (e.g., through training)? Are there conflicts of interest or loyalty? Where can I get the information? What do I need to complete the task? How can I improve communication? It is important to give all those involved sufficient time. Changes involving long-standing thought processes, evaluations, and behaviors cannot take place overnight [52].

Company physicians can co-supervise interventional approaches: in the case of diminishing strength and control with regard to workload, they can advise employers regarding reorganizing the internal balance of power [50].

### Strengths and Limitations of the Study

The study obtained data on the burnout risk and personality traits of a sample of paramedics from the Ukrainian city of Kharkiv and compared these results, obtained during the third SARS-CoV-2 wave, with data from various surveys conducted during the prepandemic period. Data from Ukraine during the pandemic period were not previously available and are thus new. Overall, the sample size was small. The extension of a local study to the whole of Ukraine should be viewed very cautiously from a statistical point of view. A larger study is needed. The paper version of the questionnaire may lead to responses of social desirability. Whether the pandemic increases the prevalence of burnout risk and whether sex-related differences in the expression of burnout symptoms actually exist have not yet been investigated.

Efforts to eliminate potential sources of bias in the study were addressed through several key measures. First, the study focused on a gender comparison to identify gender differences in burnout and personality traits, thus helping to mitigate gender bias in the interpretation of results. Second, the use of standardized instruments, such as the Maslach Burnout Inventory (MBI) and the Freiburg Personality Inventory (FPI), ensured objective data collection and minimized subjective bias. In addition, participation in the study was voluntary, reducing potential bias from pressure or coercion. These measures helped to ensure that the results of the study were as unbiased and valid across gender groups as possible.

## 5. Conclusions

The study highlights that although the prevalence of burnout among Ukrainian paramedics during the pandemic was relatively low compared to international data, it remains a significant concern. Both male and female paramedics showed moderate-to-high levels of emotional exhaustion and cynicism, highlighting the need for targeted workplace-based interventions. Future research should focus on longitudinal studies to better understand the dynamics of burnout in this group, particularly the role of personality traits in mediating stress and mental health outcomes.

## Figures and Tables

**Table 1:** Data (mean MW ± standard deviation SD) on sociodemographic and occupational data in the total sample and in the gender groups.

Variable	Total n = 88	Women n = 46	Men n = 42	*p*Mann–Whitney
	Average ± Standard Deviation			
Age (years)	35.1 ± 13.50	35.5 ± 12.92	34.7 ± 14.25	0.592
Professional years (years)	11.9 ± 12.14	11.9 ± 12.33	11.8 ± 12.07	0.973
Weekend working days (days/month)	3.6 ± 2.34	3.2 ± 2.15	3.5 ± 2.55	0.375

**Table 2:** Distribution of occupation-related data (number (%)) in the total sample and in the gender groups.

Occupational Data	Total n = 88	Women n = 46	Men n = 42	*p*Fisher
Activity				
Doctor	39 (44.3%)	20 (43.5%)	19 (45.2%)	0.868
Field shearer	49 (55.7%)	26 (56.5%)	23 (54.8%)	
Management				
No	82 (93.2%)	42 (91.3%)	40 (95.2%)	0.465
Yes	6 (6.8%)	4 (8.7%)	2 (4.8%)	
Number of working hours per week				
<12 h/wk	2 (2.3%)	0 (0%)	2 (4.8%)	0.080
12–24 h/wk	0	0 (0 5%)	0	
25–48 h/wk	1 (1.1%)	0 (0%)	1 (2.4%)	
49–72 h/wk	1 (1.1%)	0 (0%)	1 (2.4%)	
72–96 h/wk	6 (6.8%)	1 (2.2%)	5 (11.9%)	
>96 h/wk	78 (88.6%)	45 (97.8%)	33 (78.6%)	

**Table 3:** Distribution of information on sociological data (number (%)) in the total sample and in the gender groups.

Sociological Data	Total n = 88	Women n = 46	Men n = 42	*p*Fisher
Marital status				0.210
Single	39 (44.3%)	19 (41.3%)	20 (47.6%)	
Married	38 (43.2%)	18 (39.1%)	20 (47.6%)	
Widowed	4 (4.5%)	3 (6.5%)	1 (2.4%)	
Divorced	7 (8.0%)	6 (13.0%)	1 (2.4%)	
Partnership				0.440
No	33 (37.5%)	19 (41.3%)	14 (33.3%)	
Yes	55 (62.5%)	27 (58.7%)	28 (66.7%)	
Children				0.272
No	47 (53.4%)	22 (47.8%)	25 (59.5%)	
Yes	41 (46.6%)	24 (52.2%)	17 (40.5%)	
Care of family members				0.632
No	75 (85.2%)	40 (87.0%)	35 (83.3%)	
Yes	13 (14.8%)	6 (13.0%)	7 (16.7%)	

**Table 4:** MBI dimensions (MW ± SD; [points]) in the total sample and in the gender groups.

MBI Dimension	Total n = 88	Women n = 46	Men n = 42	*p*Mann–Whitney
	Average ± Standard Deviation Median (Min–Max) [95% CI]			
Emotional exhaustion	2.61 ± 1.612 (0–5.8)	2.70 ± 1.563 2.4 (0.2–5.6) [2.240–3.169]	2.51 ± 1.676 2.3 (0–5.8) [1.992–3.037]	0.572
Cynicism	1.76 ± 1.380 (0–6.0)	1.54 ± 1.463 1.2 (0–6.0) [1.109–1.978]	2.01 ± 1.256 2.0 (0–5.0) [1.613–2.396]	0.042
Performance	5.07 ± 1.208 (0–6.0)	5.08 ± 1.347 5.58 (0–6.0) [4.683–5.483]	5.06 ± 1.052 5.17 (0.5–6.0) [4.736–5.391]	0.217
MBI total score	1.85 ± 0.977 (0.20–4.32)	1.82 ± 1.014 1.55 (0.20–4.04) [1.519–2.121]	1.89 ± 0.944 1.84 (0.38–4.32) [1.594–2.182]	0.764

**Table 5:** Distribution of subjects with different levels of MBI dimensions (number (%)) in the total sample and in the respective gender groups.

MBI Dimensions	Expression (Range of Points)	Total n = 88	Women n = 46	Men n = 42	*p*Fisher
Emotional exhaustion	Low (≤2.00)	36 (40.9%)	19 (41.3%)	17 (40.5%)	0.559
	Average (2.01–3.19)	19 (21.6%)	8 (17.4%)	11 (26.2%)	
	High (≥3.20)	33 (37.5%)	19 (41.3%)	14 (33.3%)	
Cynicism	Low (≤1.00)	31 (35.2%)	22 (47.8%)	9 (21.4%)	0.027
	Average (1.01–2.19)	23 (26.1%)	11 (23.9%)	12 (28.5%)	
	High (≥2.20)	34 (38.6%)	13 (28.3%)	21 (50.0%)	
Performance capability	Low (≤4.00)	11 (12.5%)	8 (17.4%)	3 (7.1%)	0.147
	Average (4.01–4.99)	10 (11.4%)	3 (6.5%)	7 (16.7%)	
	High (≥5.00)	67 (76.1%)	35 (76.1%)	32 (76.2%)	
Burnout risk	No burnout (0–1.49)	42 (47.7%)	23 (50.0%)	19 (45.2%)	0.523
	Some burnout symptoms (1.5–3.49)	42 (47.7%)	20 (43.5%)	22 (52.4%)	
	Burnout risk (3.5–6.00)	4 (4.5%)	3 (6.5%)	1 (2.4%)	

**Table 6:** Personality traits from the FPI (stanine values as MW ± SD; [points]) in the total sample and in the gender groups.

FPI Characteristics (Stanine Values)	Total n = 88	Women n = 46	Men n = 42	*p*Mann–Whitney
	Average ± Standard Deviation Median (Min–Max) [95% CI]			
Nervousness	5.1 ± 2.22 (1–9)	5.41 ± 2.072 5.0 (1–9) [4.90–6.03]	4.69 ± 2.342 4.5 (1–9) [3.96–5.42]	0.136
Spontaneous aggressiveness	4.4 ± 2.02 (1–9)	4.41 ± 1.833 4.0 (1–9) [3.87–4.96]	4.48 ± 2.233 5.0 (1–9) [3.78–5.17]	0.864
Depressiveness	4.7 ± 2.21 (1–9)	5.11 ± 2.193 5.0 (1–9) [4.46–5.76]	4.31 ± 2.181 4.0 (1–9) [3.63–4.99]	0.152
Excitability	5.1 ± 2.14 5.0 (1–9)	5.39 ± 2.049 5.0 (1–9) [4.78–6.00]	4.83 ± 2.230 5.0 (1–9) [4.14–5.53]	0.264
Sociability	6.0 ± 1.13 6.0 (2–8)	5.98 ± 1.125 6.0 (2–8) [5.64–6.31]	6.05 ± 1.147 6.0 (2–8) [5.69–6.40]	0.761
Serenity	5.8 ± 2.15 6.0 (1–9)	5.67 ± 2.119 6.0 (1–9) [5.04–6.30]	5.88 ± 2.211 6.0 (1–9) [5.19–6.57]	0.639
Reactive aggressiveness	5.0 ± 1.91 5.0 (1–9)	4.74 ± 1.926 5.0 (1–8) [4.17–5.31]	5.38 ± 1.860 5.0 (1–9) [4.80–5.96]	0.090
Inhibition	6.1 ± 1.68 6.0 (3–9)	6.54 ± 1.486 6.0 (3–9) [6.10–6.98]	5.60 ± 1.754 6.0 (3–9) [5.05–6.14	0.025
Openness	5.3 ± 2.23 5.0 (1–9)	5.48 ± 1.997 5.0 (1–9) [4.89–6.07]	5.17 ± 2.478 5.0 (1–9) [4.39–5.94]	0.528
Extraversion	5.7 ± 1.96 (1–9)	5.70 ± 1.884 5.5 (2–9) [5.14–6.26]	5.64 ± 2.058 6.0 (1–9) [5.00–6.28]	0.993
Emotional instability	5.0 ± 2.10 (2–9)	5.30 ± 2.200 5.0 (2–9) [4.65–5.96]	4.71 ± 1.967 4.0 (2–8) [4.10–5.33]	0.221
Male/female self-portrayal	6.3 ± 1.85 (2–9)	5.78 ± 1.788 6.0 (2–8) [5.25–6.31]	6.79 ± 1.802 8.0 (2–9) [6.22–7.35]	0.009

**Table 7:** Correlation links between personality traits and burnout dimensions in the total sample.

Method/Variable		MBI			
		Emotional Exhaustion	Cynicism	Performance	Total Score
FPI	Nervousness	0.437 (<0.001)	0.439 (<0.001)	---	0.521 (<0.001)
	Spontaneous aggressiveness	0.237 (0.026)	0.304 (0.004)	---	0.271 (0.011)
	Depressiveness	0.460 (<0.001)	0.455 (<0.001)	---	0.511 (<0.001)
	Excitability	0.365 (<0.001)	0.386 (<0.001)	---	0.446 (<0.001)
	Sociability	---	---	---	0.231 (0.030)
	Serenity	−0.241 (0.024)		---	−0.269 (0.011)
	Reactive aggressiveness	---	0.269 (0.011)	---	---
	Inhibition	0.322 (0.002)	0.338 (<0.001)	---	0.398 (<0.001)
	Openness	---	---	---	0.228 (0.033)
	Extraversion	---	---	---	---
	Emotional instability	0.484 (<0.001)	0.455 (<0.001)	---	0.530 (<0.001)
	Male/female self-portrayal	---	---	---	---

Note: MBI = Maslach Burnout Inventory; FPI = Freiburg Personality Inventory. Spearman’s rho (ρ) effects were assessed according to Cohen 1988 and are <0.1, no effect; ρ = 0.1–0.29, weak effect; ρ = 0.300 to 0.499, medium effect; and ρ ≥ 0.500 strong effect.

## Data Availability

The data can be obtained from the corresponding author.
